# Carriage* Methicillin-Resistant Staphylococcus aureus* in Poultry and Cattle in Northern Algeria

**DOI:** 10.1155/2018/4636121

**Published:** 2018-06-20

**Authors:** Saliha Bounar-Kechih, Mossadak Taha Hamdi, Hebib Aggad, Nacima Meguenni, Zafer Cantekin

**Affiliations:** ^1^Regional Veterinary Laboratory of Draa-Ben-Khedda, Tizi-Ouzou, Algeria; ^2^HASAQ Research Laboratories, High National Veterinary School of Algiers, El-Harrach, Algiers, Algeria; ^3^Laboratory of Hygiene and Animal Pathology, Institute of Veterinary Sciences, University of Tiaret, Algeria; ^4^Department of Biochemistry Microbiology, Faculty of Biological and Agronomics Sciences, Mouloud Mammeri University, Tizi-Ouzou, Algeria; ^5^Department of Microbiology, Faculty of Veterinary Medicine, Mustafa Kemal University, Serinyol, Antakya, Turkey

## Abstract

Multiresistant and especially* Methicillin-Resistant Staphylococcus aureus *(*MRSA)* poses a serious public health problem that requires their immediate identification and antibiotic resistance characteristics. In order to determine antibiotic resistance* S. aureus *poultry and bovine origin, 8840 samples were collected from slaughterhouses in the northern region of Algeria between years 2009 and 2014. 8375 samples were from an avian origin (1875 from laying hens and 6500 from broiler chickens) and the rest was from bovine origin. Bacteriological isolation and identification were made by classical culture method and antibiotic resistance patterns were determined by disc diffusion test. The prevalence of* S. aureus *was 42% in laying hens, 12% in broilers, and 55% in bovine samples. The prevalence of* MRSA* was 57%, 50%, and 31% in laying hens, broiler chickens, and bovine, respectively. While* MRSA* strains isolated from poultry showed cross-resistance to aminoglycosides, fluoroquinolones, macrolides, sulphonamides, and cyclins, those isolated from bovine also revealed similar multiresistance except for sulphonamide. This high percentage of methicillin resistance and multidrug resistance in* S. aureus* poultry and bovine origin may have importance for human health and curing of human infections.

## 1. Introduction

Antibiotic resistance is prevalent in all parts of the world. Antibiotic resistance developed by bacteria is an increasingly serious threat to global public health that requires immediate actions [1]. Methicillin-resistant* Staphylococcus aureus *(MRSA) is a well-known pathogen of human and animals most commonly isolated from clinical cases [2]. MRSA was first identified in England in 1961 after which it emerged worldwide [3]. It was reported that MRSA clone ST80-IV, an epidemic community-acquired Methicillin-Resistant* Staphylococcus aureus *(C-MRSA), is widespread in the Algiers hospital and community settings [4]. Nasal carriage of multiresistant* Staphylococci* and* MRSA* in calves was detected and emphasized that they can be a cause of severe disease in humans [5]. In another study, antibiotic resistance* Staphylococci* isolated from poultry in the 1970s and 2006 was reported. Carriage of MRSA in healthy poultry was reported and emphasized that antibiotic resistance was higher in the recent isolates than prior isolates [6]. A high percentage of methicillin resistance in Staphylococci isolated from bovine mastitis was also reported in Algeria [7].

This study aimed to determine the occurrence of MRSA from laying hens, broilers, and bovine and their antibiotic resistance.

## 2. Materials and Methods

### 2.1. Study Area and Sampling

The samples from poultry and bovine were collected from different private farms of six different governorates located in northern Algeria (Bouira, Boumerdes, Tizi-Ouzou, Bejaia, Bordj-Bou-Arreridj, and Msila). Nasal swab samples were taken at the slaughterhouse after slaughtering. Total 8840 random samples were collected between 2009 and 2014; 8375 samples were from an avian origin (1875 were from laying hens and 6500 from broilers); and 465 samples were from bovine origin (Table 1).

A sterile cotton-swab was inserted as deep as possible into the nasal cavity in order to sample collection poultry. For cattle, sterile cotton-swab was inserted into the nasal cavity at a fixed distance of 10 cm to reach the ventral meatus. Each swab sample was placed in 3 ml of a transport medium (Staphylococcus enrichment broth, Axonlab) without antibiotics and transferred in an icebox. The analysis was performed the same day at the regional veterinary laboratory of the National Institute of Veterinary Medicine (Tizi-Ouzou, Algeria).

### 2.2. Bacterial Isolation and Identification

Nasal swabs were streaked onto blood agar supplemented with sheep blood 7% v/v and all inoculated media were incubated at 37°C for 24 hours as described [8, 9]. The bacterial strain purification was done on nutrient agar. Bacterial strains were identified based on the morphological criteria, biochemical characters, agglutination tests (Pastorex TM Staph-Plus kit, Bio-Rad Laboratories, France), and rabbit plasma coagulase test (Bio-Rad). All identified bacterial strains were stored -20°C in Trypticase soy broth contains 20 % glycerol.

### 2.3. Antibiotic Resistance and Susceptibility Testing

The antibiogram was performed in the same laboratory using the disk diffusion method according Clinical Laboratory Standards Institute [10] instructions in Mueller-Hinton agar (Oxoid).* S. aureus *ATCC29213 Oxacillin-sensitive strain and* S. aureus *ATCC43300 Oxacillin resistant strain were used as control strains. Twelve different antibiotic disks were used; Oxacillin (OXA, 1*μ*g), Penicillin G (PEN, 10UI), Cefoxitin (FOX, 30*μ*g), Kanamycin (KAN, 30*μ*g), Neomycin (NEO, 30*μ*g), Gentamicin (GEN, 10*μ*g), Tetracycline (TCY, 30*μ*g), Trimethoprim/Sulfamethoxazole (SXT, 1.25 *μ*g/23.75*μ*g), Enrofloxacin (ENR, 5 *μ*g), Erythromycin (ERY, 15*μ*g), and Vancomycin (VAN, 30*μ*g) (Oxoid, Hampshire, United Kingdom). Analysis of antimicrobial susceptibility test results was made by using software WHONET Version 5.6.

## 3. Results

Variable numbers and percentage of* S. aureus* were observed which were carried in laying hens, broilers, and bovines. Laying hens are shown to carry 42%, broiler chickens 12%, and bovine 55%* S. aureus *(Table 2).

Table 3 shows percentages of significant resistance and sensitivity of poultry and bovine isolated* S. aureus *against a single antibiotic.

A total of 57 % of Oxacillin resistant MRSA was observed in laying hens, 53% in broilers, and 31% in bovine. Likewise, 79 % Penicillin-resistant* S. aureus *was observed in laying hens, 92% in broilers, and 72 % in bovine. The resistance of* S. aureus *to other antibiotics is significantly mentioned in Table 3.

Interestingly and expectedly all bacterial isolates were simultaneously sensitive to Gentamicin (Table 3).

Further, multiple antibiotic cross-resistance patterns of* MRSA* identified in laying hens, broilers, and bovines were analyzed. Different nine antibiotics belonging to six different families were used, all including Oxacillin. Results shows* MRSA *strain identified in laying hens, broilers, and bovines that cross-tolerated multiple antibiotics together with Oxacillin (Tables 4, 5, and 6).

More importantly and comparatively in OXA-PEN-FOX-ERY combination,* MRSA* showed greater cross-resistivity in laying hens 33% (148/448) (Table 4) than in bovine 12% (10/80) (Table 6). Also cross-resistance of* MRSA *towards OXA-PEN-FOX-TCY combination was observed slightly less in broiler 16% (64/416) (Table 5) than in bovine 19% (15/80) as mentioned in Table 6 although highest noncomparative cross-resistance in broiler* MRSA *was 20% (82/448) towards OXA-FOX-TCY combination (Table 5) and 43% in bovine* MRSA *against OXA-PEN-FOX combination (Table 6).

## 4. Discussion

Globally* Staphylococcus aureus* bacterial pathogens are becoming resistant towards multiple antibiotics. Methicillin-resistant* Staphylococcus aureus (MRSA) *is continuously failing to be sensitive to *β*-lactam antibiotics that have caused severe problems in human and veterinary medicines. Like other countries of the world, Algeria is also facing emerging dangers posed by* S. aureus *and* MRSA* and knowledge is quite limited on the prevalence of such bacterial pathogens in Algeria compared to the world because of the lack of advanced monitoring system and tools. Although the total number of samples we analyzed from laying hens, broilers and bovine were not equal; it was observed that laying hens and broiler isolated* MRSA *strains were more resistant towards single or multiple antibiotic combinations; therefore they were cross-resistant to many other antibiotic families other than *β*-lactam antibiotics. A study carried out in eastern Algeria also showed that* MRSA* isolates in a hospital were resistant towards aminoglycosides, macrolides, fluoroquinolones, and tetracycline in many combinations. In another study in North-west of Algeria, different level of Vancomycin resistance of* MRSA *had been reported which was 54 % in poultry and 1.8 % in human [11]. In Algeria, selling antibiotics is totally uncontrolled what means abusive and indiscriminate use in human but also in the animal. Indeed, 89.09 % of the milk from 4 governorates has been found positive to antibiotics residues [12]. Moreover, 29 % of the examined milk samples produced in western Algeria contained antibacterial residues [13].

A study conducted in Egypt indicates a strong correlation between methicillin resistance and cross-resistance to *β*-lactam antibiotics [14]. Modification in Penicillin-binding protein (PLP2a) induces inactivity of Penicillin and other *β*-lactam antibiotics that develop cross-resistance of multiple antibiotics. Confirmed hospital report on* MRSA* in the human patient in Algeria increased our interest to investigate the prevalence of* S. aureus* and* MRSA* in poultry and bovine in a particular area to link with the human patient who lived in the close vicinity of such poultry and bovine animals. Of note, this antibiotic resistance data, especially on poultry, is strongly supported by previous studies like in Belgium [6] which have shown that* S. aureus* was resistant to tetracycline, macrolide, and cotrimoxazole greatly in broiler chickens. Our data on cross-resistance of MRSA in broiler chickens, towards eight antibiotics from six different families, is further supported by the previous study [5]. Multiple antibiotics cross-resistance in* MRSA* in Germany originated in broiler chicken and turkey birds [15] support our findings. However, our results show a higher prevalence of MRSA (53%) in broiler chickens than those reported which showed a prevalence of MRSA in broiler chickens (37.2%) and in turkey birds (35%). The previous study also shows the prevalence of* S. aureus* strains in poultry in Korea and Japan [5]. 44% prevalence of MRSA was also reported in poultry [14]. The prevalence of* MRSA* infections in African countries is relatively high in Nigeria, Kenya, and Cameroon, which is up to 30% and less than 10% in Tunisia [16]. In Algeria, the frequency of MRSA infection was reported to be 14 % in 2001 and recently Algerian hospitals have demonstrated a significant increase in* MRSA* that is up to 40% [4, 17]. Interestingly, MRSA incidence in French hospital is shown to be reduced [18]. Many studies suggest that farm animals show increasing trends of* MRSA* which is slightly lower in human that indicates farm animals are exposed to huge multiple antibiotics with little medical care than the humans. The emergence of MRSA came in human from swine and* mecA* gene in MRSA is predominant host restriction factors [19]. The ST398 clone has been found in different animal species including poultry and transmission of MRSA from mastitis cow was identified in a human who was working with these cows [20, 21]. Coagulase positive* S. aureus* isolates in live and slaughtered chickens were shown to be 100 % resistance towards tetracycline, Penicillin, and erythromycin and were additionally resistant to other antibiotics including Vancomycin (46.2 %) [22]. New MRSA clones and* mec* homologs among animals of the food chain and human in close contacts have been also reported [23]. The result of this study shows 57% MRSA in* S. aureus* isolated from laying hens, 53% in broiler chickens and 31% in bovine that indicates an increasing trend.

The main finding of this study clearly indicates that* S. aureus* and MRSA isolated from avian origin were more resistant to *β*-lactam antibiotics (Penicillin and Oxacillin) than bovine. This result also suggests MRSA transmission from poultry to bovine and humans that may be difficult to treat these days. In this respect, they may be a potential risk for human health.

Knowing the lack of deep research in Algeria, molecular studies are needed in order to understand the cross transmission from animal to human or from human to animals.

## Figures and Tables

**Table 1:** Sample distribution by year.

****	**Poultry**		**Bovine**
	**Laying hens**	**Broilers**	
2009	347	765	76
2010	306	970	124
2011	412	1327	85
2013	376	1785	78
2014	434	1653	102

** Total **	**1875**	**6500**	**465**

**Table 2:** Distribution of *S. aureus *and* MRSA* strain.

	**Laying hens** **n=1875**	**Broiler** **n=6500**	**Bovine** **n=465**
Nb analysed samples	1875/8840	6500/8840	465/8840
(%)	(21%)	(74%)	(5%)
Nb *S. aureus*	786/1875	789/6500	258/465
(%)	(42%)	(12%)	(55%)
Nb MRSA	448/786	416/789	80/258
(%)	(57%)	(53%)	(31%)

**Table 3:** Percentage of antibiotic resistance and sensitivity in *S. aureus*.

	**Laying hens**		**Broiler**		**Bovine**	
**Antibiotic**	**Resistance**	**Sensitive**	**Resistance**	**Sensitive**	**Resistance**	**Sensitive**
OXA	57	43	53	47	31	69
PEN	79	21	92	8	72	28
FOX	57	43	53	47	31	69
NEO	10	90	10	90	11	89
KAN	0	100	67	33	12	88
GEN	3	97	3	97	0	100
SXT	21	79	28	72	0	100
TCY	44	56	74	26	26	74
ENR	21	79	45	55	87	13
ERY	55	45	45	55	51	49
VAN	0	100	0	100	0	100

Oxacillin (OXA), Penicillin (PEN), Cefoxitin (FOX), Neomycin (NEO), Kanamycin (KAN), Gentamicin (GEN), Trimethoprim/Sulfamethoxazole (SXT), Tetracycline (TCY), Enrofloxacin (ENR), Erythromycin (ERY), and Vancomycin (VAN).

**Table 4:** Antibiotic resistance patterns of *MRSA* in hens (n=448).

**Resistance patterns**	**Number of antibiotics**	***MRSA***
OXA-PEN-FOX-ERY	3	148
OXA-PEN-FOX-TCY	4	65
OXA-PEN-FOX-ERY-TCY	5	23
OXA-PEN-FOX-SXT-TCY-ENR-ERY	7	21
OXA-PEN-FOX	3	17
OXA-PEN-FOX-TCY-ENR	5	17
OXA-PEN-FOX-SXT-TCY-ENR	6	16
OXA-PEN-FOX-NEO-SXT-TCY-ENR	7	14
OXA-PEN-FOX-NEO-SXT-TCY-ENR	7	14
OXA-PEN-FOX-SXT-TCY	5	11
OXA-PEN-FOX-NEO-TCY	5	11
OXA-PEN-FOX-SXT-TCY-ENR	6	11
OXA-PEN-FOX-SXT-TCY	5	10
OXA-FOX-TCY	3	7
OXA-PEN-FOX-SXT	4	7
OXA-PEN-FOX-TCY-GEN	5	7
OXA-PEN-FOX-TCY-NEO	4	7
OXA-PEN-FOX-ENR	4	7
OXA-PEN-FOX-SXT-ENR	5	7
OXA-PEN-FOX-NEO-SXT-TCY-ERY	7	7
OXA-PEN-FOX-TCY-ENR-ERY	6	7
OXA-PEN-FOX-NEO-GEN-SXT-TCY	7	7
OXA-PEN-FOX-NEO-GEN-SXT-TCY-ENR	8	7

**Table 5:** Antibiotic resistance patterns of *MRSA* isolated from broiler (n=416).

**Antibiotic profiles **	**Number of antibiotics**	***MRSA***
OXA-FOX-TCY	3	82
OXA-PEN-FOX-TCY	4	64
OXA-PEN-FOX-SXT	4	58
OXA-PEN-FOX	3	68
OXA-PEN-FOX-TCY	4	28
OXA-PEN-FOX-SXT-TCY	5	16
OXA-PEN-FOX-ENR-ERY	5	8
OXA-PEN-FOX-SXT-ENR	5	8
OXA-PEN- FOX-NEO-GEN-SXT-TCY	7	8
OXA-PEN-FOX-NEO-GEN-SXT-TCY-ENR	8	8
OXA-PEN-FOX-TCY-ENR-ERY	6	8
OXA-PEN-FOX-SXT-ENR-ERY	6	8
OXA-PEN-FOX-GEN-TCY-ERY	6	8
OXA-PEN-FOX-NEO-TCY	5	8
OXA-PEN-FOX-NEO-SXT-TCY-ENR	7	8
OXA-PEN-FOX-TCY-ENR	5	6
OXA-PEN-FOX-NEO-SXT-TCY-ENR	7	6
OXA-PEN-FOX-NEO-TCY	5	6
OXA-PEN-FOX-SXT-TCY-ENR	6	6
OXA-PEN-FOX-SXT-TCY	5	4

**Table 6:** Antibiotic resistance patterns of MRSA isolated from bovine (n=80).

**Antibiotic profiles**	**Number of antibiotics**	***MRSA***
OXA-PEN-FOX	3	34
OXA-PEN-FOX-TCY	4	15
OXA-PEN-FOX-ERY	4	10
OXA-PEN-FOX-NEO-KAN-TCY	6	8
OXA-PEN-FOX-ENR	4	6
OXA-PEN-FOX-ERY-ENR	5	4
OXA-PEN-FOX-NEO-KAN-TCY-ENR	7	2
OXA-PEN-FOX-ENR-ERY	5	1

## Data Availability

The data used to support the findings of this study are available from the corresponding author upon request.
